# The Interplay of Phototrophic and Heterotrophic Microbes Under Oil Exposure: A Microcosm Study

**DOI:** 10.3389/fmicb.2021.675328

**Published:** 2021-08-02

**Authors:** Manoj Kamalanathan, Kathleen A. Schwehr, Jessica M. Labonté, Christian Taylor, Charles Bergen, Nicole Patterson, Noah Claflin, Peter H. Santschi, Antonietta Quigg

**Affiliations:** ^1^Department of Marine Biology, Texas A&M University at Galveston, Galveston, TX, United States; ^2^Department of Marine and Coastal Environmental Science, Texas A&M University at Galveston, Galveston, TX, United States; ^3^Department of Oceanography, Texas A&M University, College Station, TX, United States

**Keywords:** phototrophs, heterotrophs, prokaryotes, eukaryotes, oil, photosynthesis, co-occurrence, interactions

## Abstract

Microbial interactions influence nearly one-half of the global biogeochemical flux of major elements of the marine ecosystem. Despite their ecological importance, microbial interactions remain poorly understood and even less is known regarding the effects of anthropogenic perturbations on these microbial interactions. The Deepwater Horizon oil spill exposed the Gulf of Mexico to ∼4.9 million barrels of crude oil over 87 days. We determined the effects of oil exposure on microbial interactions using short- and long-term microcosm experiments with and without Macondo surrogate oil. Microbial activity determined using radiotracers revealed that oil exposure negatively affected substrate uptake by prokaryotes within 8 h and by eukaryotes over 72 h. Eukaryotic uptake of heterotrophic exopolymeric substances (EPS) was more severely affected than prokaryotic uptake of phototrophic EPS. In addition, our long-term exposure study showed severe effects on photosynthetic activity. Lastly, changes in microbial relative abundances and fewer co-occurrences among microbial species were mostly driven by photosynthetic activity, treatment (control vs. oil), and prokaryotic heterotrophic metabolism. Overall, oil exposure affected microbial co-occurrence and/or interactions possibly by direct reduction in abundance of one of the interacting community members and/or indirect by reduction in metabolism (substrate uptake or photosynthesis) of interacting members.

## Introduction

Microbial interactions (viruses, bacteria, archaea, phytoplankton, and fungi) in aquatic systems are considered as one of the most important inter-organism associations that strongly influence carbon, nitrogen, phosphorus, sulfur, and iron cycling (Fuhrman et al., 2015). They are also the very first level of important organic matter transactions in aquatic food webs (Seymour et al., 2017). In the early 1970s, Bell and Mitchell (1972) first proposed the interaction between phytoplankton and bacteria, which could be either symbiotic or antagonistic in nature. These are now known to be integral in shaping aquatic ecosystem functions and global biogeochemistry (Ramanan et al., 2016). Similarly, phytoplankton are also infected by viruses (Pienaar, 1976) and consumed by protozoa (Caron et al., 1988) and fungi (Paterson, 1960); and often, these interactions tend to be antagonistic/parasitic in nature (Barbeau et al., 1996; Brussaard, 2004; Rasconi et al., 2011). These complex microbial relationships are also considered to be the basis of marine snow formation (Grossart et al., 2006; Azam and Malfatti, 2007), an integral mechanism in the cycling of carbon, nutrients, and trace elements in oceans (Kiørboe, 2001). Despite the significance in the aquatic ecosystems, little is known about the rates of such interactions, the composition of the molecules exchanged, the specificity of the associations, and the effects of human perturbation on these interactions and, therefore, their impact on the marine ecosystem. This knowledge gap can be primarily attributed to methodological limitations, vast spatial–temporal scales, the minute amounts of metabolites exchanged, difficulties in growing most marine microbes in the laboratory, and overall uncharacterized nature of dissolved and particulate organic matter. Although several studies have used modern omics approaches to understand these interactions, very few have focused on direct measurements (Amin et al., 2015; Durham et al., 2015; von Borzyskowski et al., 2019).

Heterotrophs derive nutrients from phytoplankton either *directly* by parasitic consumption or *indirectly* via the phytoplankton-secreted exopolymeric substances (EPSs), which are rich in polysaccharides and are broken down into simple monomers via extracellular enzymatic action (Grossart et al., 2006; Arnosti, 2011; Kamalanathan et al., 2019, 2020). A small number of examples of such interactions have been defined for bacteria and phytoplankton. For example, von Borzyskowski et al. (2019) demonstrated that certain bacteria possess several pathways to assimilate and utilize the glycolate that is widely secreted by phytoplankton. Durham et al. (2015) demonstrated the secretion of 2,3-dihydroxypropane-1-sulfonate by diatoms is assimilated and catabolized by *Roseobacter* in seawater. These interactions can also be synergistic for phytoplankton. For example, bacteria have been shown to assimilate amino acids secreted by phytoplankton and convert them into vitamin B_12_ that is released into the water, which in turn is assimilated by phytoplankton (Amin et al., 2015). Some studies also focused on indirect microbial interactions, for example, Elifantz et al. (2005, 2007) and Taylor et al. (2013), wherein labeled EPS from phytoplankton was added to a natural bacterial community. Although few in number, such detailed studies focusing on phytoplankton–protozoa and phytoplankton–fungi interactions have been virtually non-existent. In addition, the uncharacterized nature of EPS also means the possibility that numerous other pathways may exist. With increasing offshore activities, human perturbations are a great threat to aquatic ecosystems. The single-cell scale of these microbial interactions also means high vulnerability to any anthropogenic perturbations, with abiotic environmental factors previously shown to interfere with these interactions (Wang et al., 2019).

In 2010, the Deepwater Horizon oil spill exposed the Gulf of Mexico to unprecedented amounts of crude oil, resulting in oil surfacing from the wellhead to form slicks that covered a minimum area of 10,000 km^2^ of Gulf of Mexico (Hu et al., 2018). This surfacing of oil exposed microbial communities throughout the vertical water column. Several studies revealed that ∼5–31% of the total oil spilled sedimented to the seafloor (Passow et al., 2012; Fu et al., 2014; Brooks et al., 2015; Romero et al., 2015) via marine snow through a process termed Marine Oil Snow Sedimentation and Flocculent Accumulation (MOSSFA) (Daly et al., 2016; Brakstad et al., 2018; Xu et al., 2018b, 2019a). Exposure to oil induces the production of EPS as a microbial community response (e.g., Quigg et al., 2016, 2021) with significant changes to the microbial community structure (Kleindienst et al., 2015; Doyle et al., 2018). As microbial activities are an important driver in the formation of marine snow, exposure to oil is bound to alter their interactions and therefore the marine ecosystem dynamics. In addition, while the Deepwater Horizon oil spill was an extreme event, oil spills are common in the Gulf of Mexico; for example, there were more than 180 oil spills in 2019 alone^1^. Therefore, the aim of our study was to characterize microbial activities and potential microbial interactions and show how these factors are impacted by oil exposure, by combining radiotracer experiments with traditional physiological measurements (photophysiology and EPS composition analysis), microbial community composition (16S and 18S rRNA gene sequencing) and co-occurrence analysis, and a Bayesian hierarchical joint-contaminant modeling approach that combined the data from the above-mentioned analyses to identify potential interactions.

## Materials and Methods

Two sets of microcosm experiments were conducted in triplicates. The first set of microcosm experiments consisted of a short-term study lasting 8 h with three replicates of control and oil treatments with radiotracers NaH^14^CO_3_ and ^3^H-leucine and an identical set of an experiment without radiotracer additions. The other set of microcosm experiments consisted of a long-term study lasting 72 h similar to the short-term microcosm study with and without radiotracers in triplicates. Microcosm experiments were conducted using seawater collected from the Gulf of Mexico (29°22N, 93°23W), with the short-term experiment (8 h) sampled at hourly intervals of 0, 1, 2, 3, 4, and 8 h on July 18, 2019, and the long-term experiment (72 h) sampled at 0, 24, 48, and 72 h from January 22 to 25, 2019. The salinity, pH, and temperature of the seawater collected on July 18, 2019, were 25 ppt, 7.58, and 29.70°C and on January 22, 2019, were 25.66 ppt, 8.02, and 14.40°C, respectively. No nutrients were supplemented to prevent any additional alterations to the natural microbial community and activity other than those due to oil exposure. In addition, previous mesocosm experiments performed using water collected near this location did not result in nutrient limitation within 72 h (Doyle et al., 2018; Xu et al., 2018a, 2019a; Wozniak et al., 2019). For the oil treatment, Macondo surrogate oil (400 μL⋅L^–1^) was added to the seawater and mixed briefly by hand, with the same concentration used for preparation of water-accommodated fraction (WAF) of oil using the more traditional CROSERF method (Singer et al., 2001). Direct exposure to oil was chosen over the CROSERF approach to avoid missing changes to the microbial community that occur under such a procedure, which requires overnight mixing of oil with seawater (Doyle et al., 2020). Overall, each experiment was run in triplicate treatments of seawater (control) and seawater with 4 ppm of oil, containing both radiotracers NaH^14^CO_3_ and ^3^H-leucine. In addition to this microcosm, a parallel microcosm experiment was conducted in triplicates under similar conditions without the addition of radioactive tracers; and measurements including photophysiology, EPS composition, and microbial community composition (16S and 18S rRNA gene sequencing) analysis were performed during the course of these experiments. All the experiments were performed at ∼60 μmol photons⋅m^–2^⋅s^–1^ at 19°C. For both sets of the experiments, microcosm were kept under a 12:12 light:dark cycle.

### Experimental Setup With Radiotracers

The experiment was setup in 1-L culture bottles that were first cleaned with Radiac© and acid and then combusted at 450°C for 4 h. The plastic caps were fitted with two holes into which tubing was inserted and sealed into the cap with silicone. Above the cap, each tubing was fitted with a valve that remained closed during the experiment. One tubing extended to the bottom interior of the bottle and was used for withdrawing samples. The other tubing extended only into the headspace of the bottle, above the water or oil–water, and was used to apply positive pressure when withdrawing sample from the long tubing at the bottle bottom. The triplicate treatments with radiotracer additions were (1) a control with incubation of NaHC^14^O_3_ and ^3^H-leucine and (2) incubation of NaHC^14^O_3_ and ^3^H-leucine with oil. The treatments were monitored over time for radioisotope activity in different size fractions to operationally isolate eukaryotes (≥3 μm), free-living prokaryotes (<3 and ≥0.2 μm), and EPS (<0.2 μm and ≥3 kDa). We acknowledge the possibilities of prokaryotes forming aggregates, as a result being captured in 3-μm fraction, and that picoeukaryotes (with an average cellular dimensions of 3 μm or lower) are more likely to pass through this fraction. Also, there is potential for certain prokaryotes to be captured in 3-kDa fraction due to smaller cell volume than 0.2 μm or possessing non-rigid cell walls, allowing them to pass through the utilized 0.2-μm filter. However, we assume that the radiotracer signature resulting from such incidents would be relatively minimal and therefore would not affect our interpretation of results significantly.

The radiolabeled samples were taken at the specified time intervals from the two treatments and gently filtered at <100 mmHg. A 10 ml aliquot was first passed through a 3-μm filter (cellulose acetate, 25-mm diameter; Sterlitech, Kent, WA, United States). Each 3-μm filter was placed in separate 20-ml liquid scintillation vials with 5 ml of liquid scintillation cocktail added to each, and the activities of ^14^C and ^3^H retained on the 3-μm filters were counted. The 10 ml of filtrate from the 3-μm filters was then passed through a 0.2-μm filter (cellulose acetate, 25-mm diameter; Sterlitech). Each 0.2-μm filter was placed in separate 20-ml liquid scintillation vials with 5 ml of liquid scintillation cocktail added to each, and the activities of ^14^C and ^3^H retained on the 0.2-μm filters were counted. The 10 ml of filtrate from the 0.2-μm filters was then ultrafiltered through a 3-kDa membrane (EMD Amicon-15 centrifugal device; Thermo Fisher Scientific, Waltham, MA, United States), diafiltered against ultrapure 18.2 MΩ⋅cm water to remove salts, and finally made up to 1-ml volume with the ultrapure water. The 1 ml of colloidal EPS was added to 5 ml of liquid scintillation cocktail to count the activities of ^14^C and ^3^H for the 0.3 kDa colloidal EPS retentate.

Permeate (solution passing through the 3-kDa membrane) was also counted. This is the unbound fraction. An aliquot of 0.5 ml with 5 ml of cocktail was counted. The activities for ^14^C and ^3^H for each time sample for each fraction (3-μm filter, 0.2-μm filter, colloidal EPS < 0.2 μm and >3 kDa, and the unbound 3-kDa permeate) were counted and corrected to the 10-ml sample volume. The counting was done using 5 ml of Ultima Gold LLT cocktail (PerkinElmer, Waltham, MA, United States) in borosilicate glass liquid scintillation vials. The activities were counted on a Beckman Coulter (Brea, CA, United States) LS-6500 liquid scintillation counter using a dual channel program. Sample activities were corrected for isotope dilution and reagent quenching by counting a known activity spike to the same reagent matrix as the samples.

### Photo-Physiological Measurements

Photo-physiological parameters were measured in triplicate samples to monitor the effects of oil exposure on the photosynthetic activity and growth of phytoplankton. Briefly, parameters such as relative electron transport rates, light harvesting efficiency, and maximum quantum yield were measured using a Phyto-PAM fluorometer according to previous studies (Bretherton et al., 2018; Kamalanathan et al., 2019). As Phyto-PAM fluorometer uses multiwavelength excitation, the relative physiological responses of different taxa of phytoplankton were obtained using the fluorescence measurements in the channels 470 and 520 nm (diatoms and dinoflagellates); 470, 645, and 665 nm (chlorophytes); and 645 nm (cyanobacteria). The absorption cross-sectional area, connectivity factor, and Q_A_ turnover rates were measured using the Satlantic Inc. (Halifax, NS, Canada) FIRe fluorometer (Bretherton et al., 2018; Kamalanathan et al., 2019). Growth of phytoplankton was monitored by chlorophyll *a* fluorescence using Turner Designs (San Jose, CA, United States) fluorometer (Bretherton et al., 2018).

### Exopolymeric Substance Protein, Neutral Sugars, and Uronic Acid Determination

Exopolymeric substance concentration and composition were determined in triplicate samples from the parallel microcosm experiment without radiotracers. The methods used to determine protein, neutral sugars, and uronic acid in the EPS fraction are detailed in Schwehr et al. (2018) and Xu et al. (2018a). A modified bicinchoninic acid method (Smith et al., 1985) was used to determine the protein content in EPS. This was performed by using a Pierce protein assay kit (Thermo Fisher Scientific), with bovine serum albumin (BSA) as the standard. The neutral sugars concentrations were determined using the anthrone method (Morris, 1948), with glucose as the standard. This method was used for determining neutral sugars; it could not detect negatively charged sugars, such as uronic acids. Uronic acid was estimated by adding sodium borate (75 mM) in concentrated sulfuric acid and *m-*hydroxydiphenyl, with glucuronic acid as the standard (Hung et al., 2001).

### Microbial Community Composition Analysis

To elucidate the interactions between the microbes in oil and in seawater with no additions, natural seawaters from the Gulf of Mexico with their ambient communities were subsampled in triplicates from the parallel microcosm experiment without radiotracers. Throughout the experiments, the microbial assemblages were revealed using 18S and 16S rRNA sequencing, respectively. PCR amplification, using Platinum Taq DNA Polymerase (Thermo Fisher Scientific), was performed following the 16S/18S rRNA gene Illumina, Inc. (San Diego, CA, United States) amplicon protocol from the Earth Microbiome project^2^. Each sample was amplified in triplicate 25-μl reactions with the following cycling parameters: 95°C for 3 min, 30 cycles of 95°C for 45 s, 50°C for 60 s, and 72°C for 90 s, and a final elongation step at 72°C for 10 min. For prokaryotes, the V4 hypervariable region on the 16S rRNA gene and for eukaryotes the V8–V9 hypervariable region on the 18S rRNA were used for amplification (Caporaso et al., 2011; Bradley et al., 2016). For the prokaryotes, amplifications were performed using the modified 515F-806R primer pair (10 μM each) that reduces bias against the Crenarchaeota and Thaumarchaeota lineages as well as the SAR11 bacterial clade (Apprill et al., 2015; Parada et al., 2016). The primer pair utilized for the eukaryote analysis was V8f–1510r (Bradley et al., 2016). The primer pair was additionally modified to include Golay barcodes and adapters for Illumina MiSeq sequencing. Final primer sequences are detailed in Walters et al. (2016). Following amplification, the triplicate products were combined together and run on a 1.5% agarose gel to assess amplification success and relative band intensity. Triplicate amplicons were then pooled and purified with the Qiagen PCR Clean-up kit (Qiagen, Germantown, MD, United States). Approximately 50 ng of each sample was pooled; and the purified products, along with aliquots of the three sequencing primers, were sent to the Texas A&M Genomics and Bioinformatics Services (College Station, TX, United States) for MiSeq sequencing (v2 Nano chemistry, 2 × 250 bp). Sequence reads for both the 16S rRNA and 18S rRNA were processed separately using *mothur* v.1.39.5 following the MiSeq SOP https://www.mothur.org/wiki/MiSeq_SOP (Schloss et al., 2009; Kozich et al., 2013), which included reducing sequencing and PCR errors, processing the improved sequences, running an alignment using the reference SILVA alignment (v132), removing poorly aligned sequences and undesirables, and clustering utilizing the cluster.split command; and then an amplicon sequence variant (ASV) list was generated using the classify.otu command with the label “asv.” Unfortunately, not all samples gave the minimum number of reads necessary for ecological analyses. As such, one replicate from one of the control replicates at 48 h for 16s (CB3 16s), one of the control replicates at 72 h for 18s (CB4 18s), and one of the oil replicates at 0 h for 18s (OC1 18s) samples could not be used, leaving PCR amplicon duplicates for these samples. Graphical analyses were made using ggplot package (Wickham and Wickham, 2007). Diversity and richness indices (observed richness, Simpson, Shannon, and effective diversity) were calculated using the vegan package (Oksanen et al., 2013).

### Hierarchical Modeling of Species Communities

To determine the contribution of certain parameters measured in the variation of occurrence of each of the microbial species, we utilized a Bayesian hierarchical joint-contaminant modeling [hierarchical modeling of species communities (HMSC)] approach (Ovaskainen et al., 2017). We modeled the abundance of microbial species (as Y-matrix) and certain environmental parameters (treatment, time, Chlorophyll *a*, *F*_v_/*F*_m_, rETR_max_, ^14^C and ^3^H labeled >3 kDa, and >0.2- and 3-μm fraction) from the long-term experiment as the X-dataframe, using a Poisson model with a Markov chain Monte Carlo (MCMC) sampling of 10,000 iterations, 1,000 burn-in phase, 10 thinning, and the default priors of the framework.

### Co-occurrence Analysis

The co-occurrence networks for microbes from the long-term experiment were created using Spearman’s correlation matrix determined using the Hmisc package (Harrell and Harrell, 2019). The *p*-values of the correlations were then adjusted using the Benjamini and Hochberg false discovery rate (FDR)-controlling procedure (Benjamini et al., 2006), available in the Stats package. A cutoff of 0.0005 for FDR-adjusted *p*-values and 0.8 for Spearman’s rho values was then applied, and the co-occurrence was visualized using a chord diagram plotted using the package circlize (Gu et al., 2014).

### Statistics

Diversity and richness indices, HMSC modeling and co-occurrence network, and visualization were performed in R (version 4.0.2). In addition, statistical analyses of other results presented in this study such as one-way ANOVA with Tukey’s honestly significant difference (HSD), *t*-test, and Pearson’s correlation were also performed using the Vegan package (Oksanen et al., 2013) in R (R Core Team, 2013).

## Results

Two different sets of microcosm experiments with and without radiotracers were conducted in this study, one to understand the short-term (8 h) exposure of oil and the other to understand the long-term (72 h) exposure of oil. These studies were intended to capture the acute and chronic effects of exposure to oil on microbial interactions, with radiotracer study used to understand the uptake, turnover, and exchange of metabolites between phototrophs and heterotrophs, and the non-radiotracer study used to capture the physiology and community dynamics.

### Microbial Activity Signatures Using Radiotracers in the Short-Term Experiment

Monitoring of the ^14^C signature (H^14^CO_3_^–^) in the ≥ 3-μm fraction (hereafter referred to as eukaryotic phototrophic organic matter) over a period of 24 h showed no significant difference between the control and oil treatments (*p* = 0.355) (Supplementary Figure 1A). The eukaryotic phototrophic carbon label significantly increased with time in both control and oil treatments (*p* = 3.93e^–11^) (Supplementary Figure 1A). Although measurements of ^3^H-leucine in the ≥ 0.2-μm (and < 3-μm) fraction (hereafter prokaryotic organic matter) were not significantly different between the control and oil treatments for every time point (*p* = 0.311), overall, the values were higher in the control (*p* = 0.0001) (Supplementary Figure 1B). Similar to the eukaryotic phototrophic organic matter, prokaryotic organic matter also significantly increased with time (*p* < 2e^–16^) (Supplementary Figure 1B). The levels of ^14^C-labeled molecules in the ≥ 3-kDa (and < 0.2-μm) fraction (hereafter phototrophic EPS) over a period of 24 h significantly increased with time for both the treatments (*p* = 1.47e^–08^) (Supplementary Figure 1C); however, there were no significant differences over time between the control and oil treatments (*p* = 0.255) (Supplementary Figure 1C). On the other hand, the levels of ^3^H-labeled molecules in the ≥ 3-kDa (hereafter heterotrophic EPS) fraction significantly increased over 4 h but then decreased at the 8-h time point (*p* = 2.35e^–07^) (Supplementary Figure 1D), with no significant differences between the treatments (*p* = 0.814) (Supplementary Figure 1D). The levels of ^14^C in the ≥ 0.2-μm (and < 3-μm) fraction (hereafter prokaryotic uptake of phototrophic organic matter) significantly increased with time (*p* = 9.39e^–10^) (Supplementary Figure 1E); however, no significant differences were observed between the control and the oil treatments (*p* = 0.385) (Supplementary Figure 1E). Similarly, the levels of ^3^H signature in the ≥ 3-μm fraction (hereafter eukaryotic uptake of heterotrophic organic matter) increased significantly with time (*p* = 3.04e^–14^) (Supplementary Figure 1F), showing no significant differences between the treatments (*p* = 0.121) (Supplementary Figure 1F).

### Microbial Activity Signatures Using Radiotracers in the Long-Term Experiment

**FIGURE 1:**
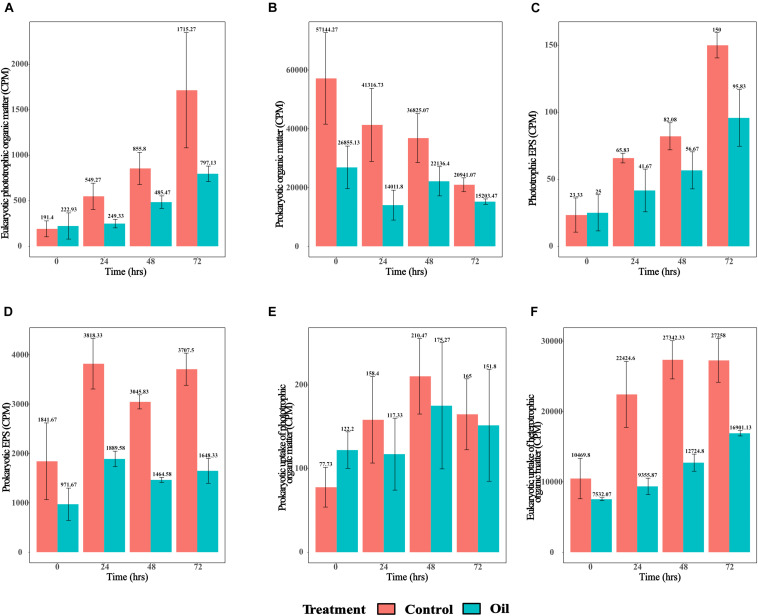
Radiotracer signals from the long-term experiment. **(A)** Eukaryotic phototrophic organic matter as indicated by the ^14^C signatures in ≥ 3-μm fraction (CPM), **(B)** prokaryotic organic matter as indicated by ^3^H signatures in ≥ 0.2-μm fraction (CPM), **(C)** phototrophic exopolymeric substance (EPS) as indicated by ^14^C signatures in ≥ 3-kDa fraction (CPM), **(D)** heterotrophic EPS indicated by ^3^H signatures in ≥ 3-kDa fraction (CPM), **(E)** prokaryotic uptake of phototrophic organic matter as indicated by ^14^C signatures in ≥ 0.2-μm fraction (CPM), and **(F)** eukaryotic uptake of heterotrophic organic matter as indicated by ^3^H signatures in ≥ 3-μm fraction (CPM).

Similar to the short-term experiment, longer-term monitoring of the eukaryotic phototrophic organic matter over a period of 72 h revealed patterns of significantly increasing labeling in both treatments with time (*p* = 1.09e^–05^) (Figure 1A). However, the levels of radiotracer signatures of ^14^C and ^3^H-labeled compounds in the control were significantly higher compared with those of the oil treatment (*p* = 0.001) (Figure 1A). Levels of prokaryotic organic matter labeling showed the opposite patterns to eukaryotic phototrophic organic matter, with decreasing activity with time in both the treatments (*p* = 0.002) (Figure 1B) and significantly higher levels of prokaryotic organic matter in the control treatments compared with those in the oil (*p* = 0.009) (Figure 1B). Labeled phototrophic EPS also increased with time for both the treatments (*p* = 9.17e^–09^) (Figure 1C), with the control treatment showing significantly higher signal than the oil (*p* = 0.0003) (Figure 1C). On the other hand, prokaryotic EPS was variable over time (Figure 1D), with a significantly higher signal in the control compared with oil treatment (*p* = 2.02e^–08^) (Figure 1D). The levels of prokaryotic uptake of phototrophic organic matter increased significantly with time for both treatments until 48 h and then decreased at 72 h (*p* = 0.034), with no differences observed between the control and oil treatments (*p* = 0.585) (Figure 1E). The eukaryotic uptake of heterotrophic organic matter increased in both control and oil treatments with time (*p* = 5.62e^–07^) (Figure 1F). However, the two treatments showed very different patterns, whereby the control had significantly higher levels of eukaryotic uptake of heterotrophic organic matter than the oil treatment (*p* = 2.70e^–08^) (Figure 1F). The levels in the control treatment peaked at 48 h and remained stable until 72 h, while this increased continuously in the oil treatment throughout the 72-h period of the experiment.

Correlation analysis between phototrophic EPS and eukaryotic uptake of heterotrophic organic matter was performed for both the short- and long-term experiments as an additional measure of phototroph–heterotroph interactions. This analysis revealed a significant linear correlation (Pearson’s *R* = 0.637, *p* = 4.279e^–06^) (Supplementary Figure 2A). Further analysis of the distribution of control and oil data points along the correlation plot revealed slightly higher distribution for control over oil; however, this difference was not statistically significant (*t*-test, *p* = 0.207) (Supplementary Figure 2B).

### Photosynthetic Physiology

**FIGURE 2:**
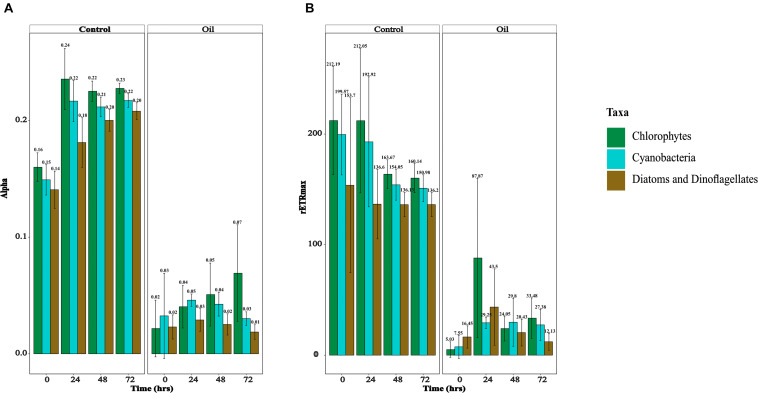
Photosynthetic physiology of different classes of phytoplankton. **(A)** Alpha/light harvesting ability (μmol e^–^⋅μmol photons^–1^) in the long-term experiment and **(B)** relative electron transport rates (μmol e^–^⋅m^– 2^⋅s^– 1^) in the long-term experiment.

Photophysiology of the phytoplankton was monitored by measuring the light harvesting ability of phytoplankton (α, μmol photons⋅μmol electrons^–1^), relative maximum electron transport rates between photosystem (PS) II and I (rETR_max_, μmol electrons⋅m^–2^⋅s^–1^), maximum quantum yield of PS II, a proxy of photosynthetic efficiency (*F*_v_/*F*_m_, relative units), connectivity between PS II units (ρ, relative units), and lastly absorption cross-sectional area of PS II, a proxy of the photosynthetic antennae size (σ, Å (quanta)^–1^) (Suggett et al., 2003). α and rETR_max_ were measured for the major taxonomic classes of phytoplankton (chlorophytes, cyanobacteria, diatoms, and dinoflagellates), whereas the other photosynthetic parameters were measured for the whole community. In the short-term experiment, although both α and rETR_max_ were not significantly different between the control and oil treatments for all taxa at every measured time point (*p* = 0.999), the overall values were higher in the control compared with the oil treatment (*p* < 9.74e^–05^) (Supplementary Figures 3A,B). In the long-term experiment, α was overall significantly higher in the control treatments compared with oil (*p* < 2e^–16^), although these differences were not significant for all time points in all taxa of phytoplankton (*p* = 0.448) (Figure 2A). Similarly, rETR_max_ values also were overall higher in the control compared with oil (*p* < 2e^–16^), but not in all taxa of phytoplankton across all time points (*p* = 0.781) (Figure 2B). It appears that diatoms and dinoflagellates had lower α and rETR_max_ than cyanobacteria, which were also less than the chlorophytes; but these were not significant (*p* = 0.192). The effects of oil were more pronounced in the long-term experiment compared with the short-term experiment. *F*_v_/*F*_m_ values were similar in the control treatment compared with oil in the short-term experiment for every time point (*p* = 0.858) (Supplementary Figure 4A), but they were only significantly higher in the long-term experiment (*p* = 0.005) (Supplementary Figure 4D). The connectivity between PS II was similar in the control and oil treatments in both the short- (*p* = 0.164) and long-term (*p* = 0.107) experiments (Supplementary Figures 4B,E). On the contrary, σ values were overall similar in the short-term experiment between the two treatments (*p* = 1.000), while they were higher in the control compared with oil treatment in the long-term experiment (*p* = 0.003); however, these were not significantly different for every measured time point (*p* = 0.238) (Supplementary Figures 4C,F).

### Exopolymeric Substances

Exopolymeric substance concentrations were monitored in the long-term experiment every 24 h by measuring the carbohydrates, proteins, and uronic acids in the ≥ 3 kDa/colloidal fraction (Supplementary Figure 5). Although an increasing trend was observed in the carbohydrate content of EPS with time in both treatments, these trends were similar between oil treatment and control (*p* = 0.189) (Supplementary Figure 4A). In addition, no significant differences were observed between the carbohydrates in the control and oil treatments (*p* = 0.277) (Supplementary Figure 5A). The protein content of EPS showed trends similar to the carbohydrate, with no significant increase in levels over time in both the treatments (*p* = 0.105) (Supplementary Figure 5B). However, protein content in the EPS of oil treatment was overall significantly higher than that in the control except for the 48-h time point (*p* = 0.003), although these differences were not significant for every measured time point (*p* = 0.098) (Supplementary Figure 5B). Uronic acid content of the EPS showed a very similar pattern to the proteins, with overall higher values in oil (*p* = 0.005), but no significant increase for every measured time point (*p* = 0.237) (Supplementary Figure 5C). Total EPS content determined by summing the carbohydrates, proteins, and uronic acids also showed trends of increasing levels with time in both the treatments, but this trend was not significant (*p* = 0.133) (Supplementary Figure 5D). However, oil treatments overall had higher levels of EPS than the control treatment (*p* = 0.039), but not for every measured time point (*p* = 0.632) (Supplementary Figure 5D).

### Microbial Community Analysis

Species richness and the Shannon–Weaver, Simpson, and effective diversity indices were similar between control and oil treatments at the starting point of the long-term experiment for both prokaryotes and eukaryotes. However, the effective diversity, Shannon–Weaver, and Simpson values were significantly lower in the oil treatment than the control at the end of the experiment for prokaryotes (*p* ≤ 0.007), while the species richness values were similar (Table 1). No such significant differences were observed for eukaryotes. While other classes of prokaryotes such as *Actinomarinales, Flavobacteriales*, *Rhodobacterales*, and *Synechococcales* were present along with Gammaproteobacteria in the control, the oil treatment was mostly dominated by Gammaproteobacteria members (Supplementary Figures 6A,B). Further analysis showed that the relative abundance of Gammaproteobacteria members *Oceanospirillales, Alteromonadales*, and *Cellvibrionales* was the highest in the oil treatments, with the relative abundance of *Oceanospirillales* increasing with time (Supplementary Figure 7B). In the control treatment, the relative abundance of *Cellvibrionales* decreased with time, while that of *Alteromonadales* increased (Supplementary Figure 7A). Interestingly, the relative abundance of *Synechococcales* was similar in the control treatment through time, while it was drastically reduced in the oil treatment after 24 h (Supplementary Figures 6A,B). Among eukaryotes, *Spirotrichea* decreased in relative abundance in both the control and oil treatments through time (Supplementary Figures 6C,D), while the abundance of *Mediophyceae* members increased with time The relative abundance of *Dinophyceae* remained considerably higher in oil, while it decreased in the control treatment through time (Supplementary Figures 6C,D). Lastly, the relative abundance of *Diatomea* and *Bacillariophyceae* members increased with time in both control and oil treatments, and the abundances were lower in the oil treatment (Supplementary Figures 6C,D).

**TABLE 1:** Spices richness and diversity of the microbial community in control and oil treatments at time 0 and 72 h of the long-term experiment.

	**Species richness**		**Shannon–Weaver**		**Simpson**		**Effective diversity**	
	**0**	**72**	**0**	**72**	**0**	**72**	**0**	**72**
**Control**								
Prokaryotes	539.75	663	3.58	5.11	0.90	0.98	36.46	168.13
Eukaryotes	756.5	341.33	4.37	3.15	0.96	0.95	79.35	24.33
**WAF**								
Prokaryotes	515	502	3.62	1.64	0.89	0.77	37.58	23.98
Eukaryotes	730	986	4.04	3.34	0.90	0.88	68.78	28.44

*WAF, water-accommodated fraction.*

**FIGURE 3:**
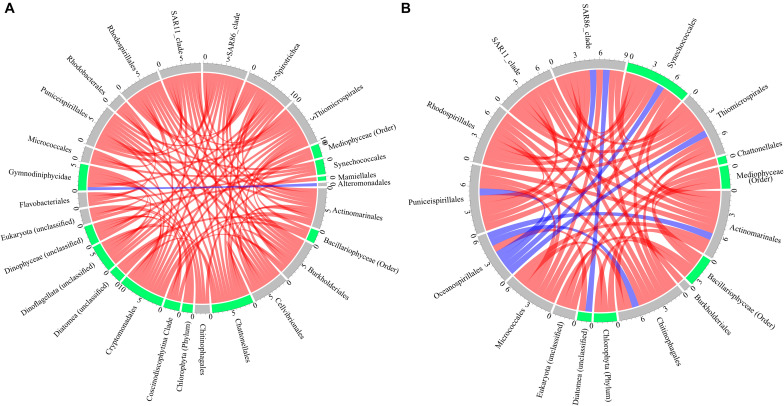
Co-occurrence of prokaryotes and eukaryotes (cutoff of false discovery rate (FDR)-adjusted *p*-values < 0.0005 and Spearman’s rho values = ± 0.8) in the **(A)** control and **(B)** oil treatments, with red color indicating positive, blue color indicating negative co-occurrence, and blue- and gray-colored tracks labeling phototrophic and heterotrophic microbes.

Positive and negative co-occurrences of species through time in the long-term experiment were determined for control and oil treatments (Figure 3). We found 89 significant co-occurrences (rho > ± 0.8, p-adjust < 0.0005) between microbial species in the control treatments, with 37 positive co-occurrences between prokaryotes, 25 positive co-occurrences between eukaryotes, and 27 co-occurrences between prokaryotes and eukaryotes (one negative and 26 positive) (Figure 3A). On the other hand, oil treatment showed only 51 significant co-occurrences (rho > ± 0.8, p-adjust < 0.0005) between microbial species with 42 significant co-occurrences between prokaryotes (6 negative and 36 positive), seven co-occurrences between eukaryotes (all 4 positive), and two co-occurrences between prokaryotes and eukaryotes (one negative and one positive) (Figure 3B).

**FIGURE 4:**
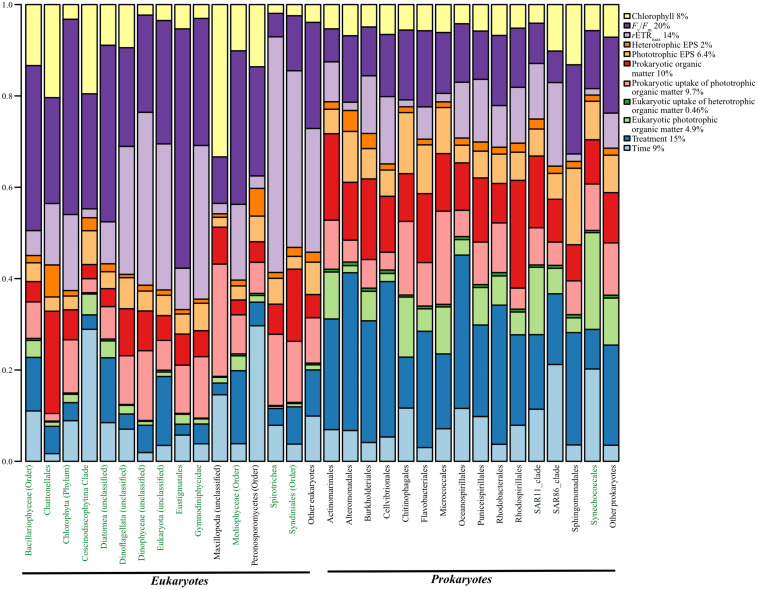
Total proportion of variation in the occurrence of prokaryotic and eukaryotic species (phototrophs marked in green) explained by various environmental parameters tested in the long-term experiment.

Lastly, we determined the amount of variation in the occurrence of microbial species accounted by the measured parameters in the long-term experiment (treatment, time, chlorophyll *a*, *F*_v_/*F*_m_, rETR_max_, and all the radiolabel measurements) using HMSC (Figure 4). Parameters such as treatment and time explain the effects of oil and changes to overall growth and oil concentration that occurred over time. Chlorophyll *a*, *F*_v_/*F*_m_, and rETR_max_ were chosen to test the effect of phytoplankton concentration, photosynthetic efficiency, and rates of photosynthesis, while parameters such as the radiolabel measurements allowed for consideration of the effects of microbial uptake of substrates and exchange of different molecules. Overall, we found that *F*_v_/*F*_m_ (photosynthetic efficiency) explained the highest amount (20%) of variation in the occurrence of microbial species, followed by treatment (15%), rETR_max_ (relative rates of photosynthesis) (14), and prokaryotic organic matter (10%) (Figure 4). On the other hand, factors such as time alone accounted for 9% of the variation, whereas both prokaryotic uptake of heterotrophic phototrophic matter and eukaryotic uptake of heterotrophic organic matter explained only 9.7 and 4.9%, respectively (Figure 4).

The variation in the occurrence of *Flavobacteriales* and *Rhodobacterales*, the two most abundant prokaryotes in the control treatment, was explained mostly by the treatment (25.4 and 30.4%, respectively). Similarly, Gammaproteobacteria members *Oceanospirillales* and *Alteromonadales*, the most abundant prokaryotes in the oil treatment, were also mostly explained by the treatment (33.5 and 34.5%, respectively) and photosynthetic efficiency (12.8 and 14.5%, respectively) (Figure 4). The variation in the occurrence of *Mediophyceae*, the most abundant eukaryote in the control as well as oil treatment, was most explained by *F*_v_/*F*_m_ (33.6%), rETR_max_ (24.1%), and treatment (15.9%) (Figure 4). Similarly, *Dinophyceae* (unclassified), the most abundant eukaryote in the oil treatment, was mostly explained by rETR_max_ (37.8%) and *F*_v_/*F*_m_ (21.2%) (Figure 4). Among the eukaryotes, the variation in the occurrence of for most phototrophs was explained by *F*_v_/*F*_m_ and/or rETR_max_. The variation in occurrence of heterotrophic eukaryote *Maxillopoda* was explained mostly by chlorophyll (33.33%), while that of *Peronosporomycetes* was explained by time (29.67%), followed by *F*_v_/*F*_m_ (23.89%). On the other hand, while the variation in the occurrence of most prokaryotes was explained by treatment (8–35%), unlike eukaryotes, the abundance of several species was explained by other factors as well. For example, *Micrococcales* and *Chitinophagales* were also explained by prokaryotic uptake of phototrophic organic matter (20.38 and 16.11%), whereas *Synechococcales* was explained by eukaryotic phototrophic organic matter (21.1%), and *Actinomarinales* and *Rhodospirillales* were also explained by heterotrophic organic matter (23.5 and 18.9%, respectively) (Figure 4).

## Discussion

Bacteria, viruses, protozoa, and fungi (heterotrophs) act as active filters between phytoplankton (phototrophs) and the rest of the marine environment, by consuming and transforming most of the organic matter, major and minor chemical elements, and energy derived by the growth of phytoplankton in the euphotic zone. These interactions between phototrophs and heterotrophs are crucial for vertical transport of nutrients in the ocean. However, the limited knowledge of these interactions makes understanding of the impacts of anthropogenic perturbations and their consequences on the biogeochemical cycles elusive. Here, we attempt to address this by specifically focusing on oil spill.

As there are uncertainties surrounding the direct estimation of primary productivity from ^14^C-bicarbonate uptake rates (Milligan et al., 2015) and bacterial productivity from ^3^H-leucine uptake rates (Franco-Vidal and Morán, 2011), we simply interpret the uptake of these radiotracers as a function of their metabolic activity. Even though there are bacteria with phototrophic and chemolithoautotrophic abilities (hence can uptake ^14^C-bicarbonate) and phototrophic eukaryotes with heterotrophic abilities (hence can uptake ^3^H-leucine), due to limitations of our experimental approach, we assume the following two scenarios for the interpretation of data from the labeling experiment: (1) uptake of radiolabeled bicarbonate was primarily carried out by phototrophs (phytoplankton and cyanobacteria) and (2) uptake of radiolabeled leucine was primarily carried out by heterotrophs and not phototrophs.

The short-term experiment with radiotracers suggested no acute effects of oil exposure on eukaryotic phototrophic uptake of inorganic carbon or secretion of organic carbon, whereas it showed slight negative effects on prokaryotic metabolic activity. Similar effects have been observed by Griffiths et al. (1981), where they observed a decrease in uptake of glucose and glutamic acid. Griffiths et al. (1981) attributed this metabolic inhibition to an event of sudden oil exposure, as an incubation over 9 days showed metabolic adjustments with higher uptake rates in their experiment. Surprisingly, these acute effects of oil on prokaryotic heterotrophic metabolism did not lead to a reduction of organic matter secretion by heterotrophs. The higher ^14^C signatures in prokaryotic fraction at 1 h and after the start of the experiment under both treatments suggested either rapid uptake of phototrophic EPS by prokaryotes or photosynthesis by smaller cyanobacteria. The earlier scenario is not surprising, as Taylor et al. (2013) previously reported incorporation of labeled EPS into the phospholipids of bacteria within 6 h. Similarly, significant increase in ^3^H signature in the eukaryotic fraction at 1 h and beyond suggests either rapid uptake of heterotrophic EPS by eukaryotes or uptake of ^3^H-leucine by prokaryotic heterotrophs residing in the phycosphere and/or *direct* uptake of ^3^H-leucine by heterotrophic eukaryotes especially protozoans or fungi (Buesing and Gessner, 2003; Matz and Jürgens, 2003). Overall, our results of microbial exchange of organic matter indicate the rapid processing of organic matter that can occur within 1 h. Moreover, the absence of any significant differences in microbial exchange of organic matter as indicated by the radiolabel signatures in the short-term experiment suggests no acute impact of oil exposure.

The long-term experiment showed severe effects of oil exposure on the uptake of radiolabeled substrates by both eukaryotic phototrophs and prokaryotic heterotrophs, with similar effects also seen for phototrophic and heterotrophic EPS secretion. However, these effects were more pronounced for prokaryotic organic matter production and the release of heterotrophic EPS. Taken together, these findings suggest that the observed severe effects on eukaryotic uptake of heterotrophic organic matter could be due to either overall lower heterotrophic radiolabeled EPS or poor performance of eukaryotic phototrophs in the presence of oil, thereby affecting the microenvironment of the phycosphere and resulting in reduced uptake of heterotrophic EPS. Interestingly, prokaryotic uptake of phototrophic organic matter was relatively unaffected in oil. As the cell diameter of *Synechococcales* (which accounted for 96.12% of the cyanobacterial community in our long-term experiment; data not shown) ranges from 0.8 to 1.2 μm (Morel et al., 1993; Garcia et al., 2016), it can occur in either filamentous or unicellular forms (Dvořák et al., 2014). Therefore, the unicellular forms of *Synechococcales* members are likely to account for the phototrophic organic matter signature in the ≥ 0.2-μm filter. However, the cyanobacterial relative abundance decreased in the presence of oil, suggesting a higher heterotrophic uptake of EPS produced from eukaryotic phototrophs.

Interestingly, a correlation of the phototrophic EPS versus eukaryotic uptake of heterotrophic organic matter (Supplementary Figure 2) showed a linear relationship, which could be interpreted as (a) evidence of metabolic dependence of prokaryotes in the phycosphere on the organic matter released by eukaryotic phototrophs and/or (b) evidence of prokaryotic organic matter uptake by phytoplankton that is linearly correlated with their organic matter secretion and/or (c) eukaryotic heterotrophic activity especially by fungi and protozoans. As it is impossible to differentiate the contribution of phototrophs from heterotrophs in our dataset, more experimentation is needed to gain further insight into this linear relationship. Regardless, the observed linear increase in the relationship with time could be due to an increase in the microbial exchange of organic matter with time, leading to an increase in intracellular accumulation of radiotracer signals, which was lower in the presence of oil. This could simply be attributed to oil toxicity and/or the competition of oil components acting as carbon source as opposed to organic matter secreted by phytoplankton.

Measurement of photosynthetic physiology suggests slight acute and severe chronic effects of oil exposure on photosynthesis of all classes of phytoplankton. This observation was surprising as there were no acute effects of oil on the uptake of ^14^C-bicarbonate seen in the eukaryotic fraction. We assume that the observed discrepancy between photosynthetic electron transport and uptake of ^14^C-bicarbonate in the eukaryotic fraction could be due to a carbon-concentrating mechanism (Raven et al., 2011) or due to the higher expenditure in the control treatment of reducing equivalents from the photosynthetic electron transport in reactions other than carbon fixation such as inorganic nitrogen assimilation or Mehler’s reaction (Raven et al., 1992; Roberty et al., 2014). Further analysis of photosynthetic physiology revealed higher connectivity of photosystems only during the short-term exposure to oil and a lower antenna size of the photosystems during the long-term exposure. Overall, consistent with previous studies (Bretherton et al., 2019, 2020), exposure to oil had differential effects on the photosynthetic physiology during short and long-term exposure, reflecting the individual- and community-level changes and acclimation induced by sudden exposure to oil.

Excess EPS production by microbes is widely considered to be a physiological response to stressful conditions such as oil exposure (Nichols et al., 2005; Quigg et al., 2016, 2021), and higher levels of EPS have been reported in the laboratory (Bacosa et al., 2018; Shiu et al., 2020), mesocosm experiments (Fu et al., 2014; Xu et al., 2018a, 2019a,b), and field observations in response to oil (Passow, 2016), whereby the protein to carbohydrate (P/C) ratio plays a critical role on EPS aggregation propensity (“stickiness”) (Santschi et al., 2020). In comparison with these observations, we found higher levels of EPS in response to oil exposure in our long-term experiment, with proteins and uronic acids contributing the most. However, the observation of higher EPS levels in the oil treatments contradicts lower levels of radiotracer signals found in the ≥ 3 kDa/colloidal fraction, suggesting that the majority of carbon especially in the protein and uronic acids of the EPS may have been derived from oil degradation. This discrepancy in our results of EPS measurements draws attention to the following limitations that cannot be directly answered in this study: (a) the contribution of organic carbon and energy from sources other than phytoplankton, i.e., oil (growth of oil-degrading bacteria and the EPS produced by them) and (b) any interaction between oil-degrading bacteria and phytoplankton.

Microbial community analysis revealed an overall decrease in prokaryotic diversity in oil treatments compared with controls as observed in several mesocosm studies (Kleindienst et al., 2015; Doyle et al., 2018). Furthermore, a higher relative abundance of eukaryotes such as *Dinophyceae, Mediophyceae*, and *Diatomea* members and prokaryotes such as oil-degrading Gammaproteobacteria members (*Oceanospirillales, Cellvibrionales*, and *Alteromonadales*) in the oil treatment are in line with the observations made in the field following the Deepwater Horizon oil spill (Karthikeyan et al., 2019; Hancock et al., 2021; Quigg et al., 2021) and also by several other mesocosm studies (Kimes et al., 2013; Kleindienst et al., 2015; Parsons et al., 2015; Almeda et al., 2018; Doyle et al., 2018; Kamalanathan et al., 2018; Bretherton et al., 2019; Gutierrez, 2019; Barbato and Scoma, 2020; Finkel et al., 2020). Assessment of co-occurrence of microbes to get insights of possible microbial interactions indicated severe effects of oil with ∼43% lower co-occurrences, which can be attributed to the observed slight decrease in microbial diversity. Interestingly, the number of negative co-occurrence was seven-fold higher in the oil treatment compared with the control. However, HMSC analysis (Ovaskainen et al., 2017) revealed factors other than treatment such as time, phototrophic EPS, heterotrophic organic matter, eukaryotic uptake of heterotrophic organic matter, and photosynthetic efficiency played an important role, suggesting a possible interaction between microbes through their activity (photosynthesis, secondary productivity, and EPS release and uptake) through time. Unsurprisingly, most of the variation in the occurrence of phototrophic eukaryotes was explained by photosynthetic parameters such as *F*_v_/*F*_m_ and rETR_max_ with an average of 28 and 21%. As *F*_v_/*F*_m_ and rETR_max_ decreased in response to oil, the effects of these two factors on microbial abundances and therefore their co-occurrences could be an *indirect* effect of oil exposure. The variation in the abundance of *Bacillariophyceae, Diatomea*, and *Mediophyceae* members were largely explained by phototrophic EPS (>24%), which along with the strong positive co-occurrence between these phototrophic species suggests a possible direct interaction between them.

Co-occurrence between prokaryotes and eukaryotes was 13-fold lower in the oil treatment compared with the control treatment. The variance in the abundance of *Chattonellales* explained by heterotrophic organic matter, together with the several significant co-occurrence with prokaryotic members such as *Cellvibrionales, Actinomarinales, Puniceispirillales*, *Rhodospirillales*, SAR11 clade, and *Thiomicrospirales* in the control treatment suggests the possibility of interaction. However, the absence of such co-occurrence with *Chattonellales* in oil can be explained by decreased abundance of the respective prokaryotic members in this treatment. Interestingly, the number of co-occurrence between the prokaryotes was nearly the same between both the treatments. *Synechococcales*, a prokaryotic phototroph showed co-occurrence with heterotrophic prokaryotes *Chitinophagales* and *Micrococcales* under both oil and control treatments. Moreover, the variation in occurrence of *Chitinophagales* and *Micrococcales* members was explained mostly by heterotrophic uptake of phototrophic organic matter and photosynthetic efficiency, suggesting (1) a possible direct interaction of *Chitinophagales* and *Micrococcales* in the phycosphere with members of *Synechococcales* and (2) that members of *Synechococcales* associated with this interaction were most likely filamentous in nature, as the variation in their occurrence was mostly explained by eukaryotic phototrophic organic matter signal that was obtained in the ≥ 3 fraction. However, further research is needed to confirm this interaction. The interaction between heterotrophic prokaryotes is, however, impossible to deduce, as the same radiolabeled substrate used was differentially assimilated by the heterotrophic prokaryotes. Regardless, treatment alone explained an average of ∼23% of the variation in the occurrence among the prokaryotes. This in combination with the reduced diversity and richness indices, which suggest a substantial effect of oil exposure on prokaryotic community and therefore their co-occurrence and/interaction with eukaryotic members. The above examples from our study indicate a reduction among several types of microbial interaction (phototrophic and heterotrophic eukaryotes, phototrophic eukaryotes and heterotrophic prokaryotes, and phototrophic and heterotrophic prokaryotes) and exchange of organic matter through EPS caused by exposure to oil.

Overall, we found that exposure to oil had a significant long-term effect on substrate uptake by phototrophs and heterotrophs, with fewer co-occurrences of microbial species and more negative co-occurrences than control. Factors such as oil exposure, photosynthesis, and prokaryotic organic matter explained a large portion of the variation in the occurrence of a microbial species and therefore how it altered microbial interactions. Lastly, our study indicates that the effect of oil exposure could be either direct by reduction of abundance of interacting members and/or indirect by reduction in metabolism (substrate uptake or photosynthesis) of interacting members.

## Data Availability Statement

The datasets presented in this study can be found in online repositories. The names of the repository/repositories and accession number(s) can be found in the article/Supplementary Material.

## Author Contributions

MK designed and performed the research, analyzed the data, and wrote the manuscript. KS designed and performed the research and analyzed the data. JL performed the research and reviewed the manuscript. CT, CB, NP, and NC performed the research. PS designed the research and reviewed the manuscript. AQ helped design the research, wrote and reviewed the manuscript, and obtained the funding. All authors contributed to the article and approved the submitted version.

## Conflict of Interest

The authors declare that the research was conducted in the absence of any commercial or financial relationships that could be construed as a potential conflict of interest.

## Publisher’s Note

All claims expressed in this article are solely those of the authors and do not necessarily represent those of their affiliated organizations, or those of the publisher, the editors and the reviewers. Any product that may be evaluated in this article, or claim that may be made by its manufacturer, is not guaranteed or endorsed by the publisher.

## Footnotes

1
https://incidentnews.noaa.gov/

2
https://earthmicrobiome.org/protocols-and-standards/empo/

## Supplementary Material

Supplementary Figure 1 Radiotracer signals from the short-term experiment. **(a)** eukaryotic phototrophic organic matter (CPM), **(b)** prokaryotic organic matter (CPM), **(c)** phototrophic EPS (CPM), **(d)** heterotrophic EPS (CPM), **(e)** prokaryotic uptake of phototrophic organic matter (CPM), and **(f)** eukaryotic uptake of heterotrophic organic matter (CPM).

Supplementary Figure 2 Phytoplankton-bacteria interaction. **(a)** Linear correlation of phototrophic EPS (CPM) vs. eukaryotic uptake of heterotrophic organic matter (CPM) in both short and long-term experiment, and **(b)** Distribution of ratio of phototrophic EPS (CPM) and eukaryotic uptake of heterotrophic organic matter (CPM) in both short and long-term experiment.

Supplementary Figure 3 Photosynthetic physiology of different classes of phytoplankton in the short-term experiment. **(a)** Alpha/light harvesting ability (μmol e^–1^. μmol photons^−1^) in the short-term experiment, **(b)** relative electron transport rates (μmol e^–^. m^–2^. s^–1^) in the short-term experiment.

Supplementary Figure 4 Photosynthetic performance and characteristics of phytoplankton. **(a)** *F*_v_/*F*_m_, maximum quantum yield (relative units) of phytoplankton in the short-term experiment, **(b)** Photosystem II connectivity (ρ, relative units) in the short-term experiment, **(c)** Sigma, absorption cross-section area of photosystem II (σ, Å (Quanta) ^–1^) in the short-term experiment, **(d)** *F*_v_/*F*_m_, maximum quantum yield (relative units) of phytoplankton in the long-term experiment, **(e)** Photosystem II connectivity (ρ) in the long-term experiment, and **(f)** Sigma, absorption cross-section area of photosystem II (σ, Å (Quanta) ^–1^) in the long-term experiment.

Supplementary Figure 5 Exopolymeric substance (EPS) composition in the long-term experiment. **(a)** Carbohydrate concentration of EPS (μg.L^–1^), **(b)** Protein concentration of EPS (μg.L^–1^), **(c)** Uronic acids concentration of EPS (μg.L^–1^), and **(d)** Total EPS concentration of EPS (μg.L^–1^).

Supplementary Figure 6 Relative ASV abundance of microbes. **(a)** Relative prokaryotic abundance in the control treatment, **(b)** Relative prokaryotic abundance in the oil treatment, **(c)** Relative eukaryotic abundance in the control treatment and **(d)** Relative eukaryotic abundance in the oil treatment through time in the long-term experiment.

Supplementary Figure 7 ASV Abundance of members belong to class Gammaproteobacteria in **(a)** the control and **(b)** the oil treatment through time in the long-term experiment.

Supplementary Table 1 Number of sequenced and processed reads and accession numbers for the 16S and 18S rRNA genes.
