# Supplementary material for: The Interplay of Phototrophic and Heterotrophic Microbes Under Oil Exposure: A Microcosm Study
Source: Front Microbiol. 2021 Aug 2;12:675328. doi: 10.3389/fmicb.2021.675328 (PMC8366316; doi:10.3389/fmicb.2021.675328)
Supplement: Supplementary Figure 1 — Radiotracer signals from the short-term experiment. (a) eukaryotic phototrophic organic matter (CPM), (b) prokaryotic organic matter (CPM), (c) phototrophic EPS (CPM), (d) heterotrophic EPS (CPM), (e) prokaryotic uptake of phototrophic organic matter (CPM), and (f) eukaryotic uptake of heterotrophic organic matter (CPM). [file Data_Sheet_1.zip › Supplementary Table 1.docx]

**Supplementary Table 1.** Number of sequenced and processed reads and accession numbers for the 16S and 18S rRNA genes on the file 675328

| **Sample ID** | **Sample** | **Raw sequences** | **After filtering and omitting redundancy (ASVs)** | **SRA sample accession number** | **SRA Bioproject accession number** |
| --- | --- | --- | --- | --- | --- |
| CA1_16SrRNA | CA1 | 30068 | 22503 | CA1_16SrRNA | SRR13287729 |
| CA2_16SrRNA | CA2 | 28621 | 18809 | CA2_16SrRNA | SRR13287728 |
| CA3_16SrRNA | CA3 | 39739 | 22437 | CA3_16SrRNA | SRR13287717 |
| CA4_16SrRNA | CA4 | 28766 | 22796 | CA4_16SrRNA | SRR13287706 |
| CB1_16SrRNA | CB1 | 27341 | 20280 | CB1_16SrRNA | SRR13287695 |
| CB2_16SrRNA | CB2 | 21584 | 12333 | CB2_16SrRNA | SRR13287677 |
| CB3_16SrRNA | CB3 | 4425 | 2452 | CB3_16SrRNA | SRR13287678 |
| CB4_16SrRNA | CB4 | 32745 | 16471 | CB4_16SrRNA | SRR13287679 |
| CBST_16SrRNA | CBST | 15457 | 9713 | CBST_16SrRNA | SRR13287680 |
| CC1_16SrRNA | CC1 | 21228 | 11839 | CC1_16SrRNA | SRR13287726 |
| CC2_16SrRNA | CC2 | 24810 | 12344 | CC2_16SrRNA | SRR13287725 |
| CC3_16SrRNA | CC3 | 15279 | 7046 | CC3_16SrRNA | SRR13287724 |
| CC4_16SrRNA | CC4 | 10592 | 4983 | CC4_16SrRNA | SRR13287723 |
| OA1_16SrRNA | OA1 | 67132 | 50849 | OA1_16SrRNA | SRR13287721 |
| OA2_16SrRNA | OA2 | 28376 | 17811 | OA2_16SrRNA | SRR13287720 |
| OA3_16SrRNA | OA3 | 38587 | 25814 | OA3_16SrRNA | SRR13287719 |
| OA4_16SrRNA | OA4 | 41042 | 28850 | OA4_16SrRNA | SRR13287718 |
| OAST_16SrRNA | OAST | 41413 | 29786 | OAST_16SrRNA | SRR13287716 |
| OB1_16SrRNA | OB1 | 19935 | 11357 | OB1_16SrRNA | SRR13287714 |
| OB3_16SrRNA | OB3 | 41651 | 27114 | OB3_16SrRNA | SRR13287713 |
| OB4_16SrRNA | OB4 | 35784 | 23926 | OB4_16SrRNA | SRR13287712 |
| OBST_16SrRNA | OBST | 44660 | 35532 | OBST_16SrRNA | SRR13287711 |
| OC1_16SrRNA | OC1 | 12613 | 7817 | OC1_16SrRNA | SRR13287709 |
| OC2_16SrRNA | OC2 | 28488 | 15848 | OC2_16SrRNA | SRR13287708 |
| OC3_16SrRNA | OC3 | 40331 | 30897 | OC3_16SrRNA | SRR13287707 |
| OCST_16SrRNA | OCST | 43543 | 30411 | OCST_16SrRNA | SRR13287705 |
|  |  |  |  |  |  |

**Supplementary Table 1.** Continued

| **Sample ID** | **Sample** | **Raw sequences** | **After filtering and omitting redundancy (ASVs)** | **SRA sample accession number** | **SRA Bioproject accession number** |
| --- | --- | --- | --- | --- | --- |
| CA1_18SrRNA | CA1 | 12099 | 10348 | CA1_18SrRNA | SRR13287704 |
| CA2_18SrRNA | CA2 | 27483 | 23925 | CA2_18SrRNA | SRR13287703 |
| CA3_18SrRNA | CA3 | 13393 | 11969 | CA3_18SrRNA | SRR13287702 |
| CA4_18SrRNA | CA4 | 1633 | 1075 | CA4_18SrRNA | SRR13287701 |
| CAST_18SrRNA | CAST | 292 | 240 | CAST_18SrRNA | SRR13287684 |
| CB1_18SrRNA | CB1 | 28569 | 24494 | CB1_18SrRNA | SRR13287700 |
| CB2_18SrRNA | CB2 | 12168 | 10667 | CB2_18SrRNA | SRR13287699 |
| CB3_18SrRNA | CB3 | 42281 | 36699 | CB3_18SrRNA | SRR13287681 |
| CB4_18SrRNA | CB4 | 1369 | 158 | CB4_18SrRNA | SRR13287682 |
| CBST_18SrRNA | CBST | 32866 | 24915 | CBST_18SrRNA | SRR13287727 |
| CC1_18SrRNA | CC1 | 18337 | 15863 | CC1_18SrRNA | SRR13287683 |
| CC2_18SrRNA | CC2 | 17320 | 15531 | CC2_18SrRNA | SRR13287685 |
| CC3_18SrRNA | CC3 | 62552 | 58109 | CC3_18SrRNA | SRR13287686 |
| CC4_18SrRNA | CC4 | 40904 | 33026 | CC4_18SrRNA | SRR13287687 |
| CCST_18SrRNA | CCST | 39750 | 29134 | CCST_18SrRNA | SRR13287722 |
| EB_18SrRNA | Control | 28179 | 25055 | EB_18SrRNA | SRR13287675 |
| PCR_18SrRNA | ExtBlank | 8627 | 741 | PCR_18SrRNA | SRR13287676 |
| OA1_18SrRNA | OA1 | 16216 | 12272 | OA1_18SrRNA | SRR13287688 |
| OA2_18SrRNA | OA2 | 66055 | 57437 | OA2_18SrRNA | SRR13287689 |
| OA3_18SrRNA | OA3 | 55455 | 47569 | OA3_18SrRNA | SRR13287690 |
| OA4_18SrRNA | OA4 | 57852 | 47166 | OA4_18SrRNA | SRR13287691 |
| OAST_18SrRNA | OAST | 51246 | 39376 | OAST_18SrRNA | SRR13287715 |
| OB1_18SrRNA | OB1 | 15443 | 13102 | OB1_18SrRNA | SRR13287692 |
| OB2_18SrRNA | OB2 | 27301 | 23491 | OB2_18SrRNA | SRR13287693 |
| OB3_18SrRNA | OB3 | 46149 | 40796 | OB3_18SrRNA | SRR13287694 |
| OB4_18SrRNA | OB4 | 55348 | 45276 | OB4_18SrRNA | SRR13287696 |
| OBST_18SrRNA | OBST | 33044 | 25885 | OBST_18SrRNA | SRR13287710 |
| OC1_18SrRNA | OC1 | 461 | 369 | OC1_18SrRNA | SRR13287697 |
| OC2_18SrRNA | OC2 | 24100 | 21365 | OC2_18SrRNA | SRR13287698 |
| OC3_18SrRNA | OC3 | 57622 | 50033 | OC3_18SrRNA | SRR13287671 |
| OC4_18SrRNA | OC4 | 69277 | 49243 | OC4_18SrRNA | SRR13287672 |
| OCST_18SrRNA | OCST | 39961 | 33915 | OCST_18SrRNA | SRR13287673 |
|  |  |  |  |  |  |
|  |  |  |  |  |  |
| PCR_18SrRNA | ExtBlank |  |  | PCR_18SrRNA | SRR13287676 |
| EB_16SrRNA | ExtBlank | 15395 | 13843 | EB_16SrRNA | SRR13287674 |
