# Supplementary figures and images for: The Interplay of Phototrophic and Heterotrophic Microbes Under Oil Exposure: A Microcosm Study
Source: Front Microbiol. 2021 Aug 2;12:675328. doi: 10.3389/fmicb.2021.675328 (PMC8366316; doi:10.3389/fmicb.2021.675328)

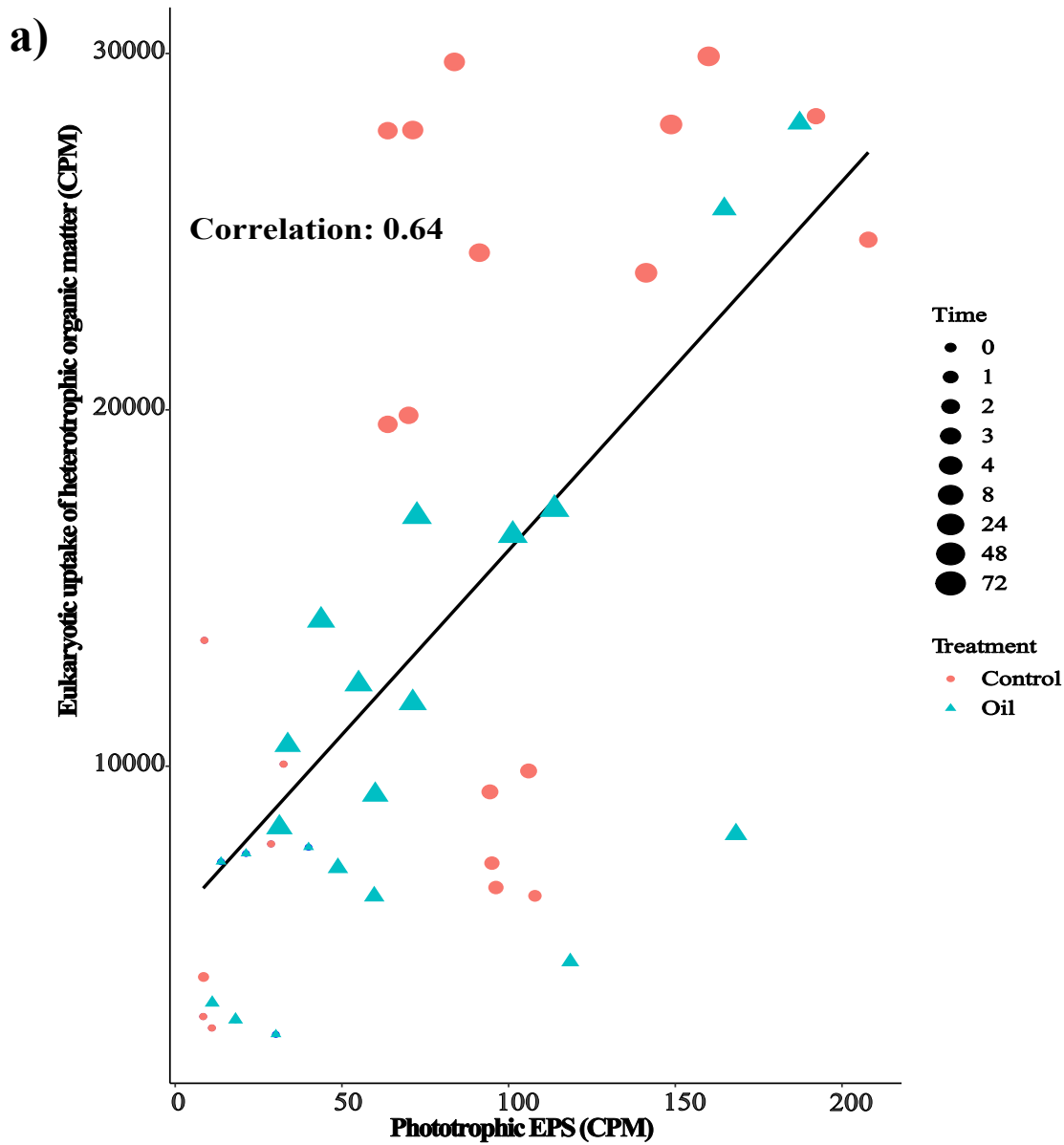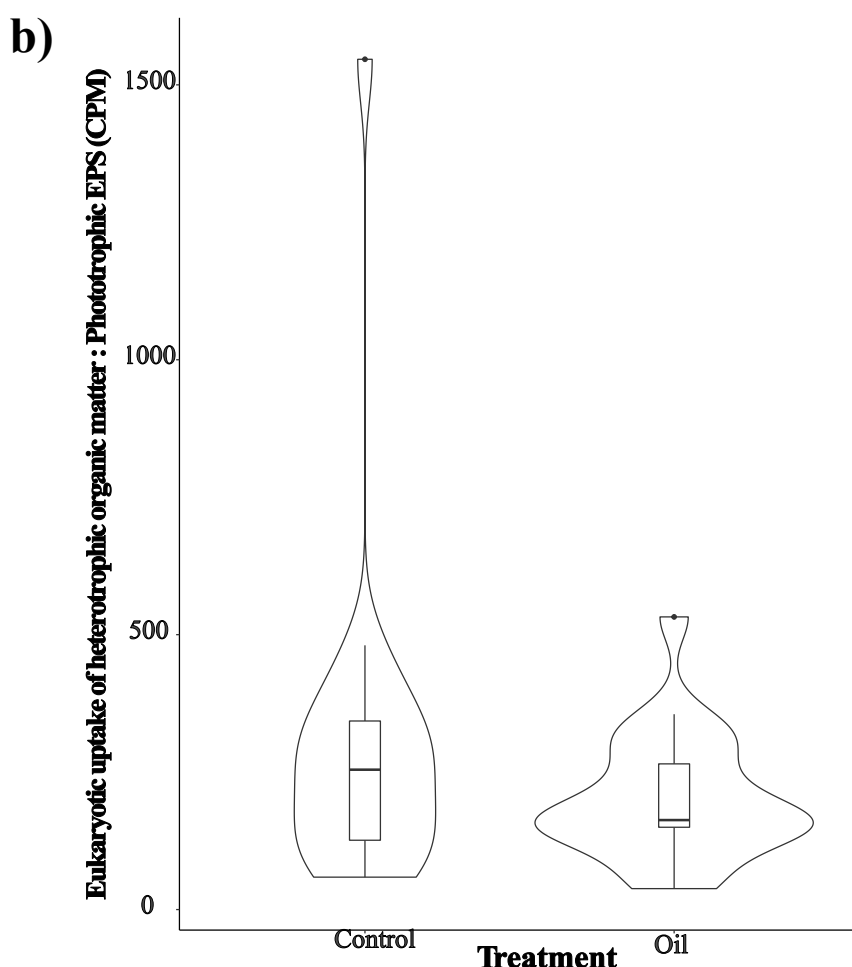

Supplement: Supplementary Figure 1 — Radiotracer signals from the short-term experiment. (a) eukaryotic phototrophic organic matter (CPM), (b) prokaryotic organic matter (CPM), (c) phototrophic EPS (CPM), (d) heterotrophic EPS (CPM), (e) prokaryotic uptake of phototrophic organic matter (CPM), and (f) eukaryotic uptake of heterotrophic organic matter (CPM). [file Data_Sheet_1.zip › Supplementary Figure 2.pdf]

a)

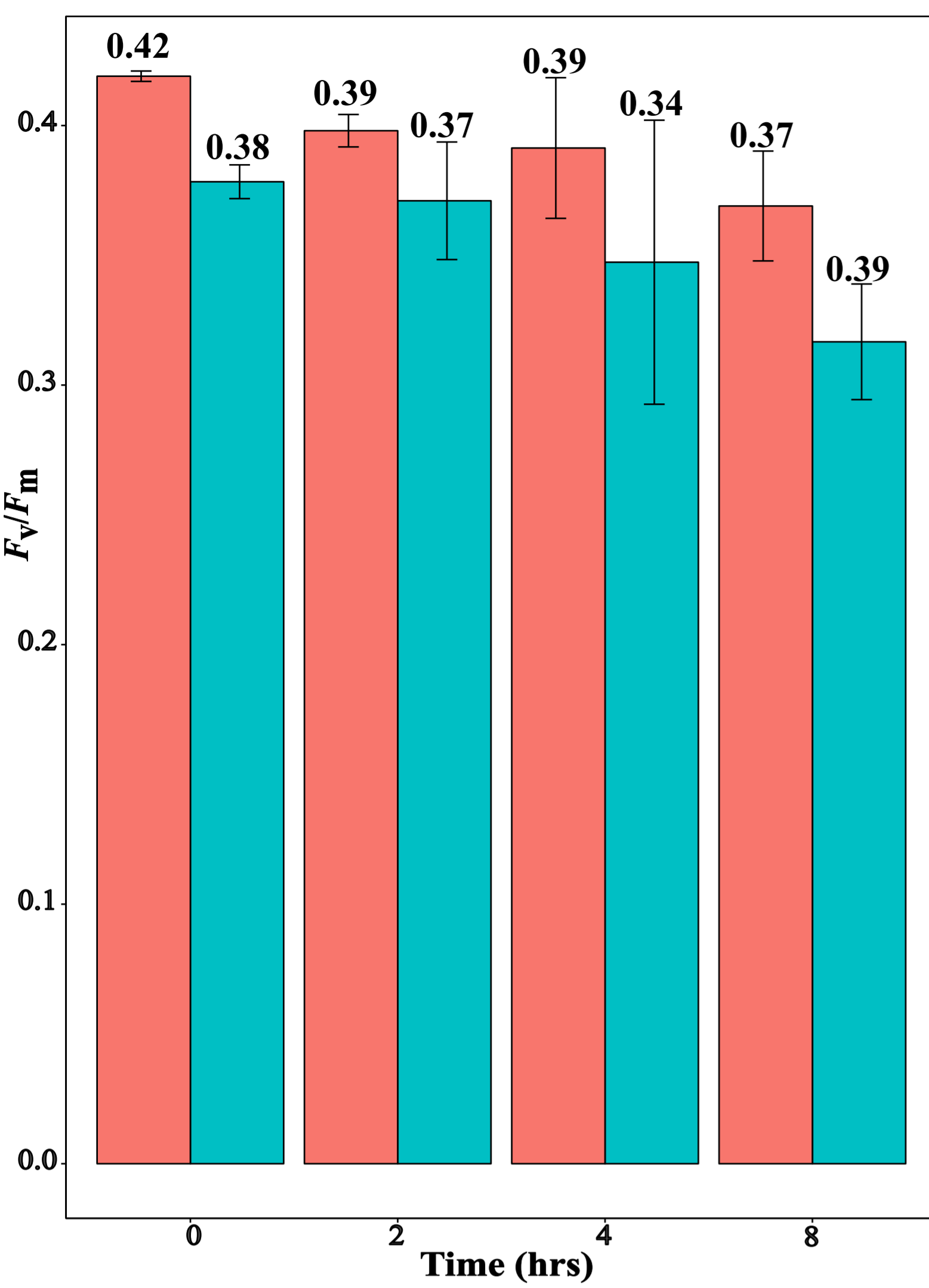

b)

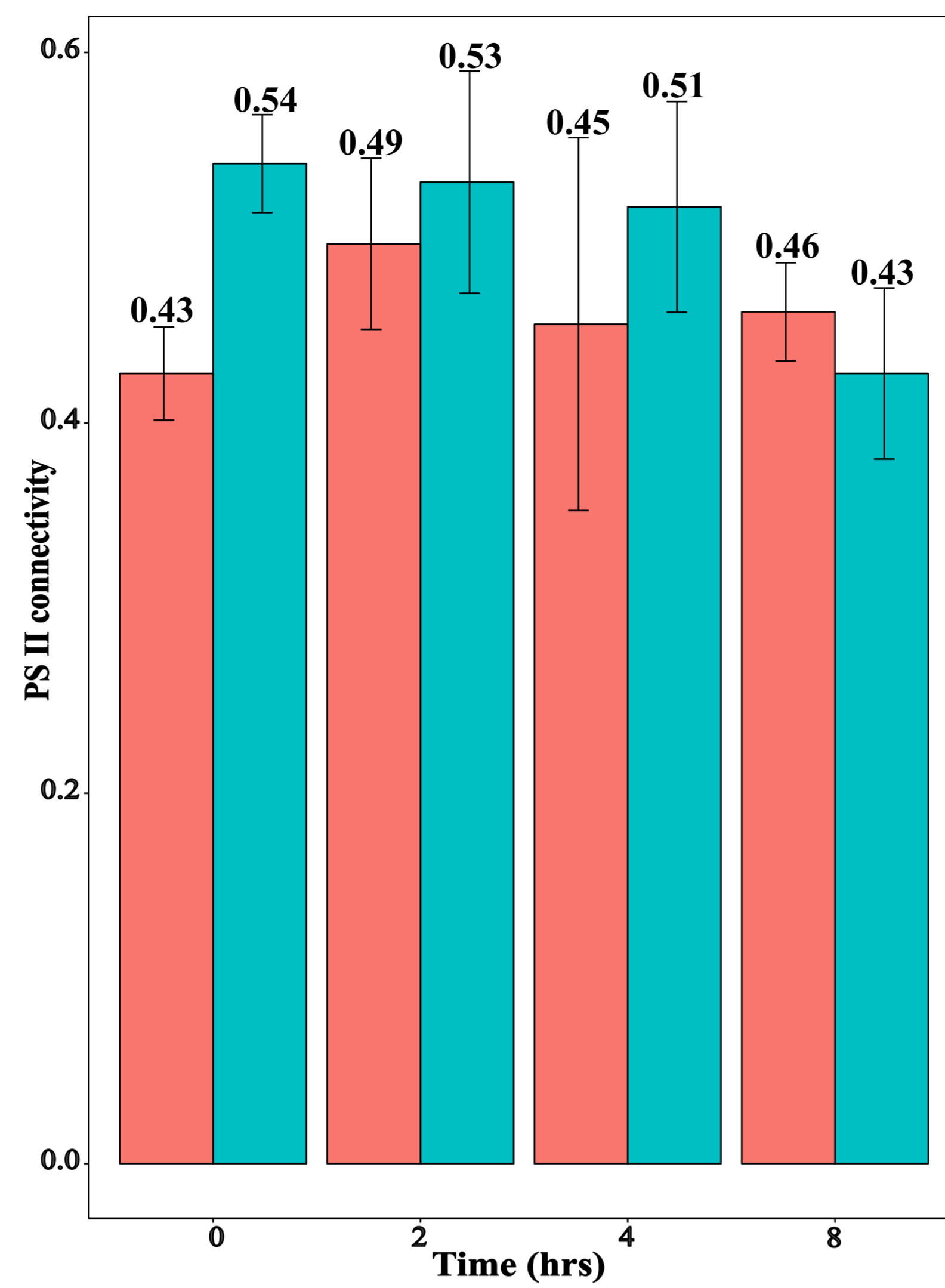

c)

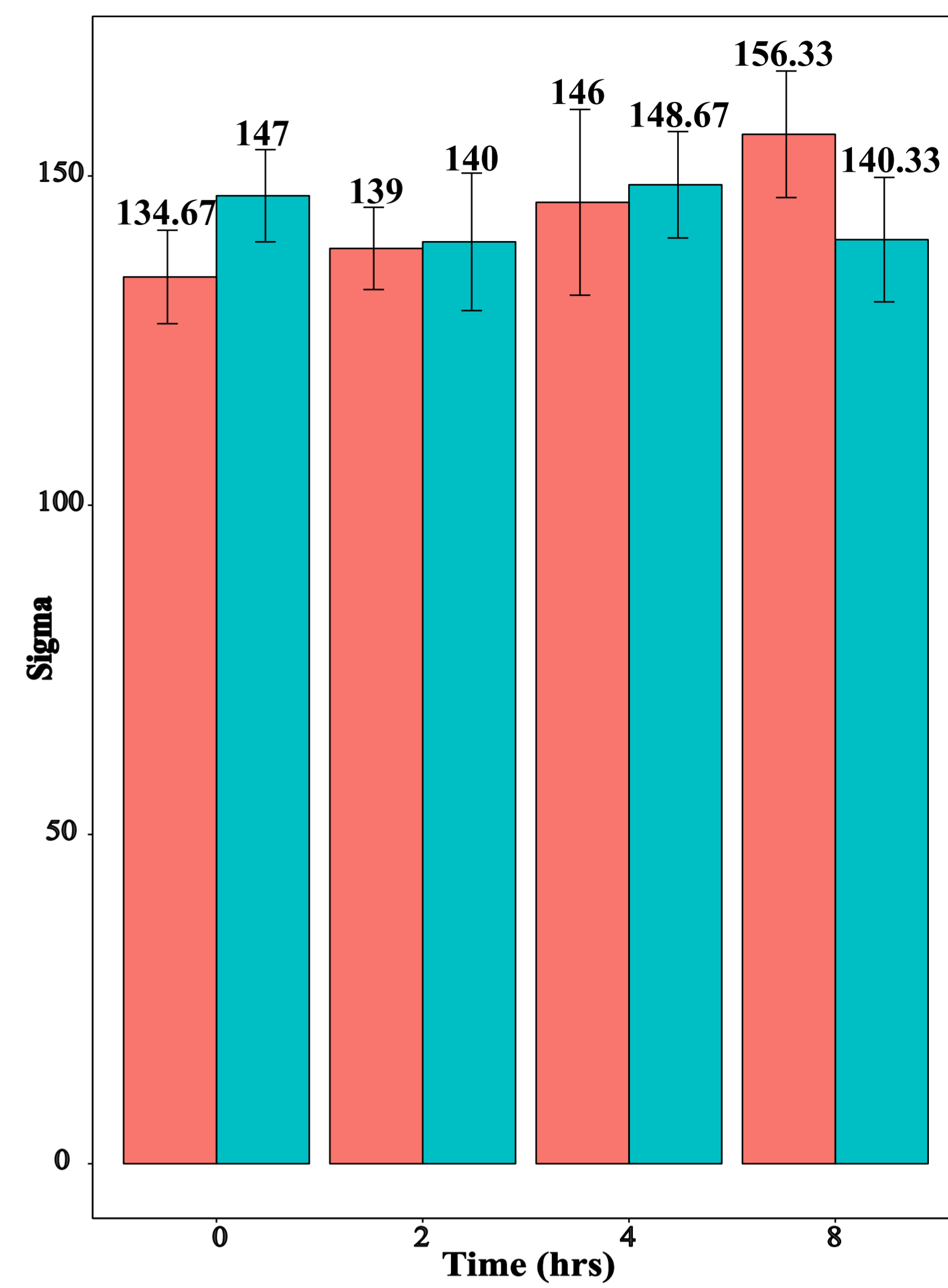

d)

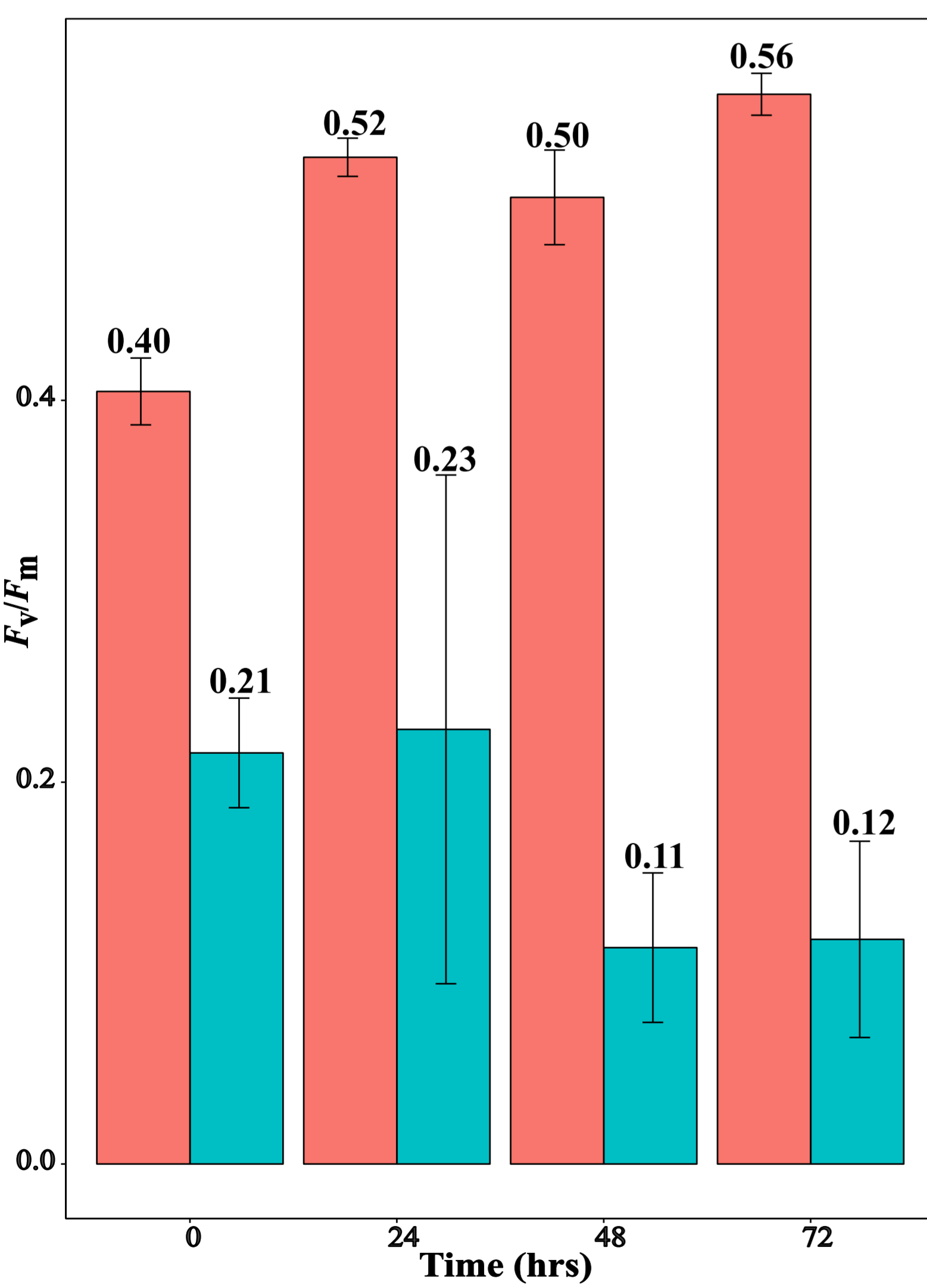

e)

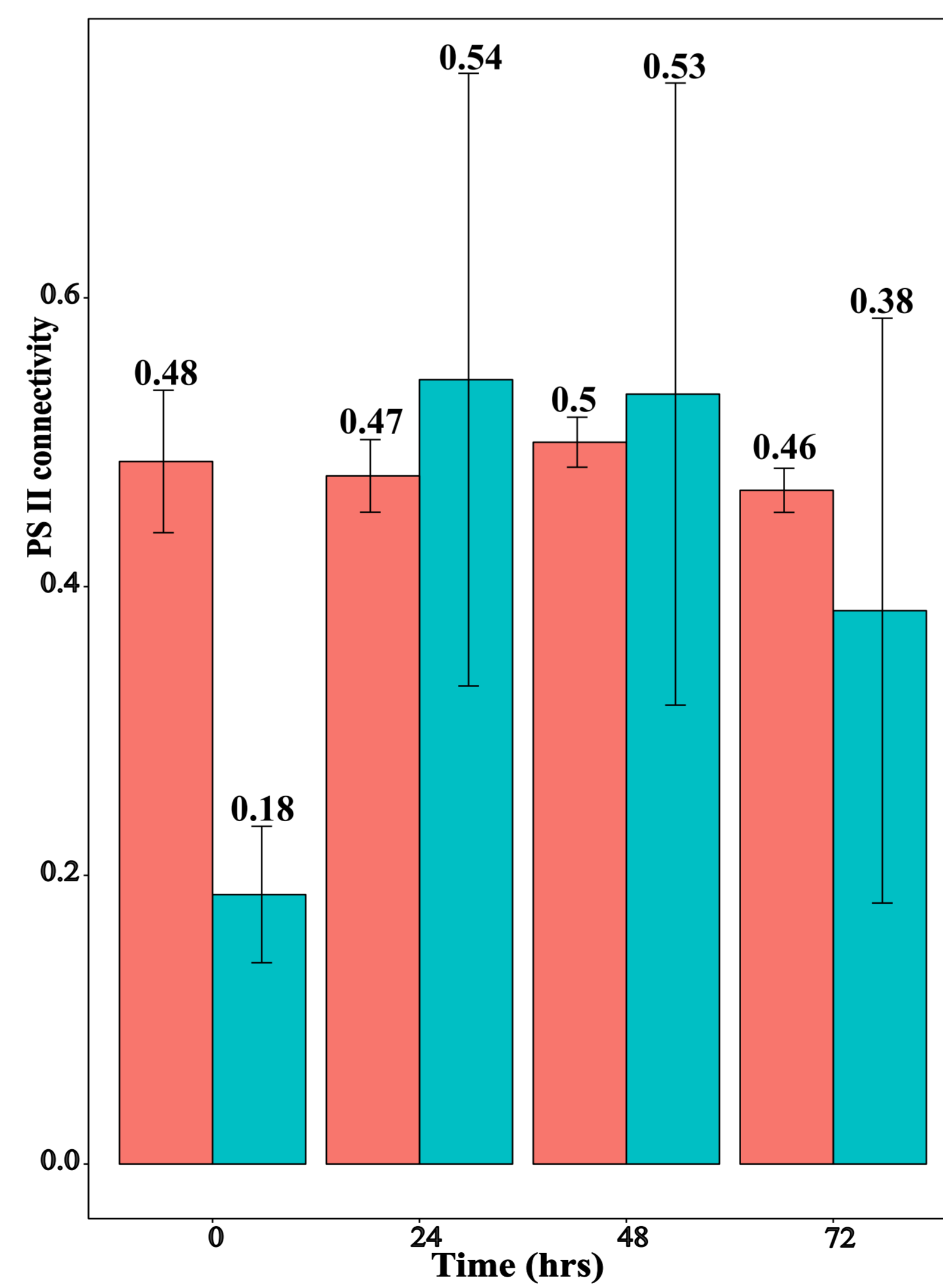

f)

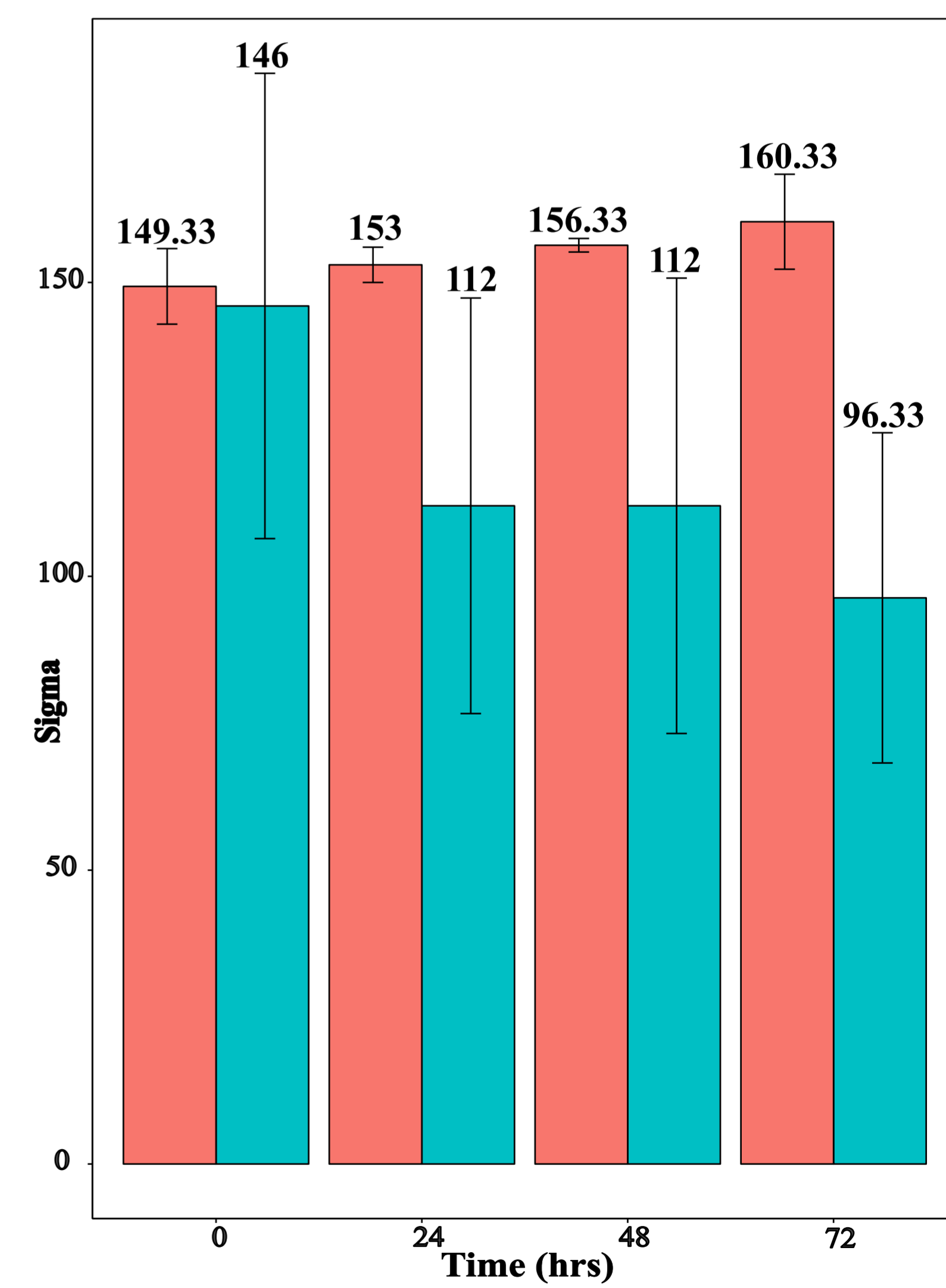

Treatment    Control    Oil

Supplement: Supplementary Figure 1 — Radiotracer signals from the short-term experiment. (a) eukaryotic phototrophic organic matter (CPM), (b) prokaryotic organic matter (CPM), (c) phototrophic EPS (CPM), (d) heterotrophic EPS (CPM), (e) prokaryotic uptake of phototrophic organic matter (CPM), and (f) eukaryotic uptake of heterotrophic organic matter (CPM). [file Data_Sheet_1.zip › Supplementary Figure 4.pdf]

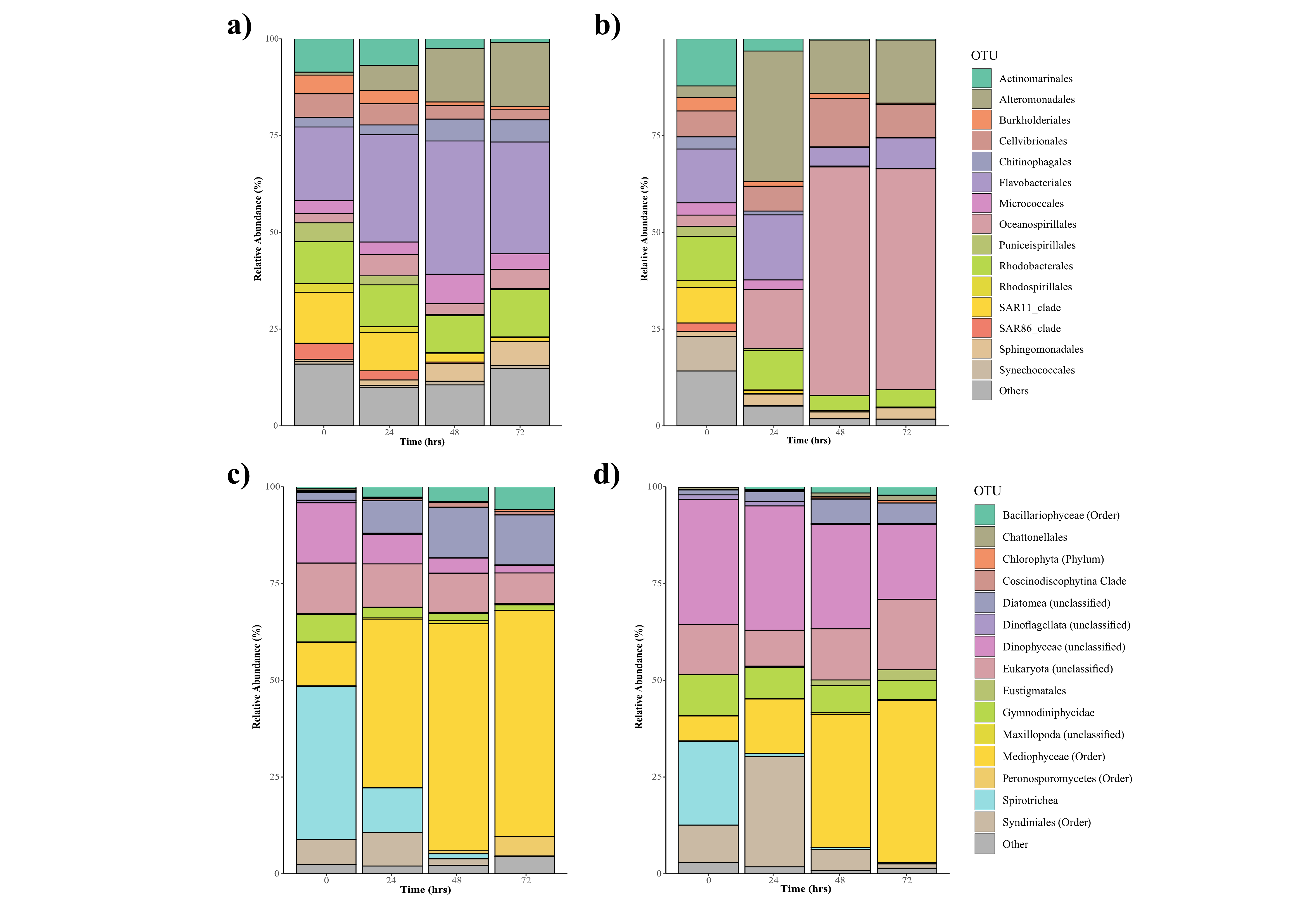

Supplement: Supplementary Figure 1 — Radiotracer signals from the short-term experiment. (a) eukaryotic phototrophic organic matter (CPM), (b) prokaryotic organic matter (CPM), (c) phototrophic EPS (CPM), (d) heterotrophic EPS (CPM), (e) prokaryotic uptake of phototrophic organic matter (CPM), and (f) eukaryotic uptake of heterotrophic organic matter (CPM). [file Data_Sheet_1.zip › Supplementary Figure 6.tiff]

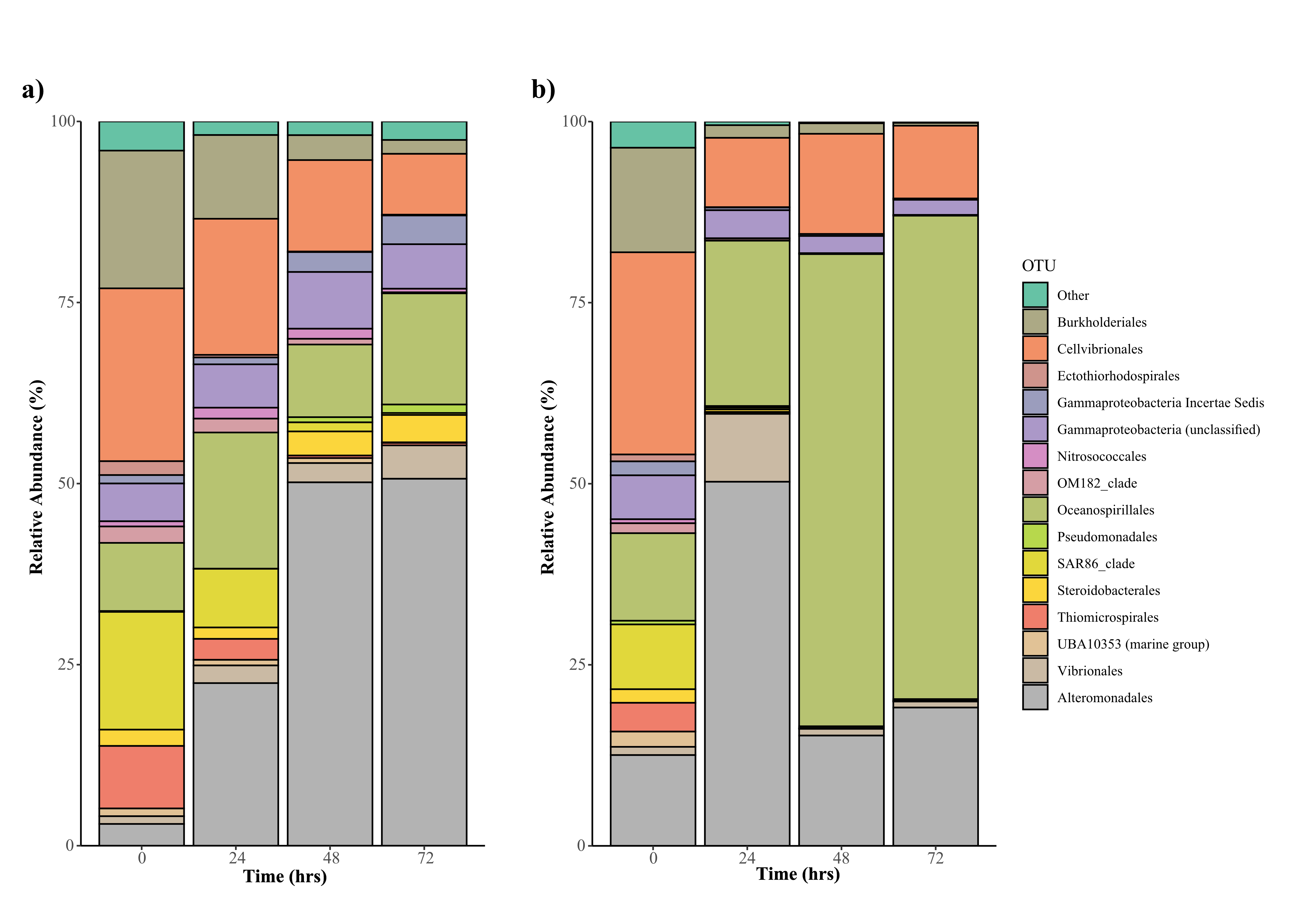

Supplement: Supplementary Figure 1 — Radiotracer signals from the short-term experiment. (a) eukaryotic phototrophic organic matter (CPM), (b) prokaryotic organic matter (CPM), (c) phototrophic EPS (CPM), (d) heterotrophic EPS (CPM), (e) prokaryotic uptake of phototrophic organic matter (CPM), and (f) eukaryotic uptake of heterotrophic organic matter (CPM). [file Data_Sheet_1.zip › Supplementary Figure 7.tiff]
